# Prevalence and Clinical Conditions Related to Sarcopaenia among Older Persons Living in the Community

**DOI:** 10.3390/jcm11133814

**Published:** 2022-07-01

**Authors:** Encarnación Blanco-Reina, Ricardo Ocaña-Riola, Gabriel Ariza-Zafra, María Rosa García-Merino, Lorena Aguilar-Cano, Jenifer Valdellós, Claudia Torres-Blanco, Inmaculada Bellido-Estévez

**Affiliations:** 1 Pharmacology and Therapeutics Department, Instituto de Investigación Biomédica de Málaga-IBIMA, School of Medicine, University of Málaga, 29016 Málaga, Spain; ibellido@uma.es; 2 Escuela Andaluza de Salud Pública, 18011 Granada, Spain; ricardo.ocana.easp@juntadeandalucia.es; 3 Geriatrics Department, Complejo Hospitalario Universitario, 02006 Albacete, Spain; gariza@sescam.jccm.es; 4 Health District of Málaga-Guadalhorce, 29009 Málaga, Spain; rosaballet@yahoo.es (M.R.G.-M.); jenny_dok7@hotmail.com (J.V.); 5 Physical Medicine and Rehabilitation Department, Hospital Regional Universitario, 29010 Málaga, Spain; loagca2011@hotmail.com; 6 Pharmacology and Therapeutics Department, School of Medicine, University of Málaga, 29016 Málaga, Spain; claudiatblanco4@gmail.com

**Keywords:** sarcopaenia, older adults, primary care, psychopathology, frailty, body mass index

## Abstract

(1) Background: In health care and in society at large, sarcopaenia is a disorder of major importance that can lead to disability and other negative health-related events. Our study aim is to determine the prevalence of sarcopaenia among older people attended in primary care and to analyse the factors associated with this age-related clinical condition; (2) Methods: A multicentre cross-sectional study was conducted of 333 community-dwelling Spanish adults aged 65 years or more. Sociodemographic, clinical, functional, anthropometric, and pharmacological data were collected. Sarcopaenia was defined following European Working Group on Sarcopaenia in Older People (EWGSOP) criteria; (3) Results: Sarcopaenia was present in 20.4% of the study sample, and to a severe degree in 6%. The intensity of the association between sarcopaenia and frailty was weak-moderate (Cramer V = 0.45). According to the multinomial logistic regression model performed, sarcopaenia was positively associated with age and with the presence of psychopathology (OR = 2.72; 95% CI = 1.30–5.70) and was inversely correlated with body mass index (OR = 0.73, 95% CI = 0.67–0.80; (4) Conclusions: Sarcopaenia commonly affects community-dwelling older persons and may be associated with age, body mass index, and psychopathology. The latter factor may be modifiable or treatable and is therefore a possible target for intervention.

## 1. Introduction

In recent years, there has been growing interest in identifying age-related conditions that can lead to disability. In this context, special attention has been paid to the study of sarcopaenia, a condition that is closely related to physical function impairment. The term sarcopaenia was originally proposed in 1989 to describe age-related decrease in muscle mass [1,2]. Subsequently, various operational definitions and diagnostic criteria have been proposed. It has been suggested that defining sarcopaenia only in terms of muscle mass is of very limited value, for several reasons. Firstly, the association between this criterion alone and adverse health outcomes is weak. Moreover, muscle strength does not depend exclusively on muscle mass, and the relationship between them is not linear [3,4]. In response to these observations, a functional dimension has been added to the term. In 2010, the European Working Group on Sarcopaenia in Older People (EWGSOP) provided a working definition for sarcopaenia [5], proposing that it be diagnosed using the twin criteria of low muscle mass and low muscle function (either low strength and/or low physical performance). This operational definition represented an important change and is currently in wide use, worldwide. More recently, however, in order to reflect the growing body of new scientific and clinical evidence regarding this question, the EWGSOP recommendations have been updated (as EWGSOP2) [6], and the broad description of sarcopaenia is that it is a muscle disease (or failure) rooted in adverse muscle changes that accrue over a lifetime. In fact, sarcopaenia is already formally recognised as a muscle disease, with a specific ICD-10-MC diagnosis code, which represents an important step forward towards a generally accepted classification [7,8].

Progressive muscle loss in the elderly is due in part to physiological age-related changes, such as the loss of motoneuron units, decreased hormone status, and increased insulin sensitivity, which in turn lead to increased proteolysis, decreased muscle protein synthesis, and increased fat infiltration of the muscle [9]. Other factors that may contribute to the development of sarcopaenia include immobility, inflammation, an inactive lifestyle, and malnutrition. We believe it important to seek a better understanding of this geriatric syndrome due to its prevalence, its association with negative health-related events, the existence of certain potentially reversible factors, and because it is, at least initially, a treatable condition. In this respect, physical exercise to gain muscle resistance and a focused nutritional intervention are of fundamental importance.

Widely varying accounts have been given of the prevalence of sarcopaenia [10,11,12,13,14]. In part, this is because it depends on the characteristics of the population under study (such as age, gender, comorbidity, and race) and on the healthcare setting considered, but the value obtained is also subject to the methodology used to assess muscle mass and even to the definition made of sarcopaenia [11]. Even when focusing exclusively on community-dwelling older people, the heterogeneity of the samples analysed, the criteria used, and the cut-off points selected combine to affect the prevalence obtained, reported values of which range from 6% to 59.8% [11], or from 9.9% to 40.4% according to other sources [12,13,14]. In any case, the global rate of sarcopaenia is undoubtedly rising across the world, and the impact is especially high among the elderly population in nursing homes and those who are hospitalised or in rehabilitation units [11].

Sarcopaenia has a major impact on society and its healthcare systems, imposing severe personal, social, and economic burdens [15]. Among other consequences, it increases the risk of falls and fractures [16,17], impairs patients’ ability to perform activities of daily living [18], provokes mobility disorders [19], limits independence [20], decreases the quality of life [21], and can even lead to death [22,23].

In view of these considerations, a heightened awareness of the dangers of sarcopaenia should become a part of clinical routine, with special regard to community-dwelling older persons. Primary care is the most common health contact point for most of the older population. Moreover, attention is usually comprehensive and personalised, making this healthcare environment very suitable for the identification, management, and study of sarcopaenia. However, the assessment and treatment of sarcopaenia at the outpatient level is still uncommon. Accordingly, this complex syndrome is probably underdiagnosed [24]. In view of its relationship with disability and other negative health-related events, and the need to continue characterising its prevalence in different settings, the present study was designed to address these questions. The progression of sarcopaenia depends on various factors, and their joint effects are directly relevant to the possibilities of prevention and treatment [25]. The main aim of the present study is to determine the prevalence of sarcopaenia among older adults living in the community in Spain, where life expectancy rates are among the highest in the world [26], and to analyse related factors, some of which are potentially modifiable through specific interventions and preventive actions.

## 2. Materials and Methods

This multicentre cross-sectional study was carried out within a primary healthcare context.

### 2.1. Reference Population and Study Sample

The study sample was composed of persons aged 65 years or more, living in the community, attended at primary care centres in Malaga (Spain). All participants were registered in the database of the Spanish NHS, were treated in an outpatient setting (i.e., not institutionalised), and provided signed informed consent to take part in the study. As exclusion criteria, none had implanted metal devices (pacemakers or osteoarticular prostheses, because they might cause interference with electric bioimpedanciometry measurements), had suffered the complete or partial amputation of a limb, or had an advanced or terminal illness. The patients were recruited at nine primary healthcare centres, using stratified random sampling designed to obtain a representative sample. The study population was allocated in proportion to the size of each healthcare centre. Based on a published prevalence of sarcopaenia in primary care of 22% [27,28] and assuming a margin of error of less than 4.5% and a 95% confidence interval, we calculated that the minimum sample size required for this study would be 325 persons.

### 2.2. Data Collection and Global Assessment

The participants were interviewed using a structured questionnaire, and various physical tests were conducted to assess the presence and degree of sarcopaenia. Further data were obtained from medication packaging and digital medical records. A complete set of sociodemographic, clinical, functional, and pharmacological data were collected from all participants. The clinical data included all diseases recorded, possible comorbidities, and Charlson’s comorbidity index (CCI) [29]. Information was also obtained about the medication prescribed (indication, dosage, and duration of any treatment received during the last three months or more). Polypharmacy was defined as the chronic prescription of five or more drugs.

Cognitive function was evaluated using Pfeiffer’s short portable mental state questionnaire (SPMSQ) [30], and mood status was determined using Yesavage’s geriatric depression scale (GDS-15) [31]. The patients’ independence in performing instrumental activities of daily living (IADL) was assessed using the Lawton scale [32]. The body mass index (BMI) was calculated, and nutritional screening was performed using the Spanish version (Nestlé Nutrition Institute) of the Mini Nutritional Assessment-Short Form [33]. The different BMI categories (underweight, normal, overweight, and obesity) were operationalised following the World Health Organization cut-off values.

Frailty was assessed according to the phenotype proposed by Fried et al. [34], which consists of the following criteria: (a) unintentional weight loss of 4.5 kg or more in the previous year; (b) self-reported exhaustion, identified by two questions on the Center for Epidemiological Studies Depression (CES-D) scale; (c) weakness, defined by low handgrip strength and measured in kg in the dominant hand using a dynamometer (highest of three consecutive measurements), adjusted for gender and BMI (grip strength was classified as low when the force exerted was below the first quintile of the distribution); (d) slow walking speed (lowest quintile of gait speed), assessed by the walking time (in seconds) over a distance of 4.57 m, adjusted for gender and height; and (e) low physical activity, measured by the weighted score of kilocalories expended per week, obtained from the Minnesota Leisure Time Activity Questionnaire and adjusted for gender. Participants were classified as non-frail (robust) if they met none of the criteria, pre-frail if they met one or two criteria, and frail if three or more criteria were met.

### 2.3. Assessment of Sarcopaenia

The main study outcome was sarcopaenia, which was defined following EWGSOP criteria [5]. According to these criteria, diagnosis of sarcopaenia required the documentation of low muscle mass plus the documentation of either low muscle strength or low physical performance.

#### 2.3.1. Muscle Mass

Muscle mass was measured by bioelectrical impedance analysis (BIA). The BIA resistance (measurement range 150–1200 Ohms) was determined using a Tanita BC-418 body composition analyser (Tanita Corporation, Tokyo, Japan) with an 8-electrode method and an operating frequency of 50 kHz. Muscle mass was calculated using Janssen’s bioelectrical impedance analysis equation [35]. Absolute skeletal muscle mass was converted to skeletal muscle index (SMI) by dividing the limb skeletal muscle mass (kg) by the square of the height (m^2^). Low muscle mass was defined as the SMI of two standard deviations (SDs) or more below the normal sex-specific mean for young persons. Using the cut-off points indicated in the EWGSOP consensus, low muscle mass was classified as SMI < 8.87 kg/m^2^ in men and <6.42 kg/m^2^ in women.

#### 2.3.2. Muscle Strength

Muscle strength was assessed by grip strength, measured using a Jamar hydraulic grip hand dynamometer SP-5030J1 (Lafayette Instrument Company, Lafayette, IN, USA). Patients were instructed to perform a maximal isometric contraction, and the highest value of three consecutive measurements was recorded. BMI-adjusted values were used as cut-off points to classify low muscle strength (following EWGSOP recommendations for men/women) [5].

#### 2.3.3. Physical Performance

Usual walking speed (m/s) on a 4-metre course was used as an objective measure of physical performance. The time elapsed from the start to the finish point was recorded by an investigator with a digital chronometer, and the best time of two attempts was recorded. A cut-off point of 0.8 m/s or less in gait speed was used to define low physical performance [5].

Sarcopaenia was diagnosed as follows: low muscle mass alone was defined as pre-sarcopaenia; the joint presence of low muscle mass and low muscle strength or low performance was defined as sarcopaenia; and the presence of all three criteria was considered as severe sarcopaenia.

All data were measured and collected by primary care clinicians, who were active members of the research team.

### 2.4. Statistical Analysis

Exploratory data analysis and frequency tables were used to describe the study variables. Taking into account the four possible categories of the main variable according to the EWGSOP conceptual stages of sarcopaenia (pre-sarcopaenia, sarcopaenia, severe sarcopaenia, and no sarcopaenia), a multinomial logistic regression model was used to study the relationship between the independent variables and the outcome variable, sarcopaenia [36]. All independent variables were included in the regression model. The influence of various factors on the states of pre-sarcopaenia, sarcopaenia, and severe sarcopaenia was examined, taking non-sarcopaenic patients as a benchmark. Odds ratios (OR) and 95% confidence intervals (CI) were calculated for each covariate included in the model. A 5% significance level was assumed to indicate statistical significance. All statistical data analyses were performed using SPSS version 24.0 (IBM SPSS Statistics, Armonk, NY, USA).

### 2.5. Ethical Considerations

This study was conducted in accordance with the Declaration of Helsinki. The Málaga Clinical Research Ethics Committee approved the study (PI-0234-14), and informed consent was obtained from all patients prior to their inclusion.

## 3. Results

### 3.1. Characteristics of the Study Population

The study population consisted of 333 community-dwelling Spanish adults aged 65 years or more. Their mean age was 72.81 years (standard deviation 5.1, range 65–91), and slightly more than half were female. Only 19.5% lived alone; the rest lived with a partner, family member(s), or caretaker (professional or otherwise). The average CCI score was 1.30 (standard deviation 1.4, range 0–7), and 33.3% of the patients had a score > 2. Each patient presented an average of 7.4 diagnoses (standard deviation 3.4, range 0–20) and was consuming 6.5 drugs (standard deviation 4.0; range 0–21), with a polymedication prevalence of 65.8%. The most prevalent chronic conditions were bone and joint disorders (mainly osteoarthritis of the knee, hip, hand, and shoulder) (76.9%), hypertension (68.2%), and dyslipidaemia (51.7%). Some form of psychopathology (mainly anxiety and/or depression) was present in 37.8% of the patients, and 42.6% suffered insomnia. The mean score on the Lawton scale was 6.7 (standard deviation 1.7, range 0–8) with half of the sample being independently capable of performing IADL. Regarding anthropometric and nutritional characteristics, the mean BMI was 30.3 kg/m^2^ (standard deviation 4.9, range 18.9–52.3). Only 14.7% of the patients had a normal weight; 39.6% presented overweight and 45.6% obesity. Among the participants with obesity, more than half (56.6%) were class I (30–34.9 kg/m^2^), 34.9% were class II (35–39.9 kg/m^2^), and 8.5% were obesity class III or severe (>40 kg/m^2^). Nevertheless, according to the MNA, 95.5% of the participants had a good nutritional status and only 3.3% were at risk of malnutrition or were malnourished (1.2%). The sociodemographic, functional, cognitive, and clinical characteristics of the study participants are summarised in Table 1.

Frailty was present in 21.3% of participants; 57.1% were pre-frail and 21.6% were non-frail. The most prevalent Fried phenotype criterion observed in the sample was weakness (62.8%), followed by low physical activity (49.2%) and exhaustion (20.4%).

### 3.2. Assessment of Sarcopaenia and Related Factors

According to the EWGSOP algorithm, 20.4% of the community-dwelling older adults in the sample had sarcopaenia, and 6% had severe sarcopaenia. Slightly more than half (57.7%) of the participants did not meet any criteria for sarcopaenia and 15.9% were considered pre-sarcopaenic (low muscle mass alone) (Table 2). The mean SMI was 7.6 kg/m^2^ (standard deviation 1.4; range 4.8–12.0). For the female participants, the mean SMI was 6.7 kg/m^2^ (standard deviation 0.8; range 4.8–9.2), and for the men, it was 9.0 kg/m^2^ (standard deviation 0.9; range 5.8–12.0). Therefore, sarcopaenia was present, overall, in 26.4% of this elderly population. The condition was more common in women (29.2%) than in men (22.5%), and among the non-obese than the obese (37% vs. 13.8%, respectively; *p* < 0.001). The coincidence of obesity and sarcopaenia was present in 6.3% of the sample. In this respect, too, the mean BMI was higher in non-sarcopaenic than in sarcopaenic patients (31.3 kg/m^2^ versus 27.6 kg/m^2^, respectively; *p* < 0.001).

Regarding the combination of sarcopaenia and frailty, 7.8% of participants were both frail and sarcopaenic, while 13.5% were frail-only. None were sarcopaenic-only. Therefore, there were no patients who were sarcopaenic and robust at the same time, and all sarcopaenic individuals were either in a state of pre-frailty (70.5%) or one of frailty (29.5%). The prevalence of frailty among those with sarcopaenia was 40.2%; among those with frailty, the prevalence of sarcopaenia was 36.6%. The intensity of the association between sarcopaenia and frailty was weak-moderate (Cramer V = 0.45).

A multinomial logistic regression analysis was performed to further examine the influence of the independent variables on the EWGSOP sarcopaenia categories (Table 3). The two factors that were most consistently associated with the presence of sarcopaenia were BMI and the diagnosis of a psychopathology. In fact, the odds of presenting sarcopaenia and severe sarcopaenia decreased by 27% and 25% for each additional point (kg/m^2^) of BMI (OR = 0.73, 95% CI = 0.67–0.80; OR = 0.75, 95% CI = 0.66–0.86), respectively. However, these odds rose sharply for persons with a psychopathology, for all states of sarcopaenia. Thus, the OR of patients with vs. without a psychopathology were 2.56 (95% CI = 1.06–6.19) for pre-sarcopaenia, 2.72 (95% CI = 1.30–5.70) for sarcopaenia, and 7.89 (95% CI = 2.25–27.59) for severe sarcopaenia, with all other covariates being equal. No relevant association was found between sarcopaenia and the other prevalent pathologies considered or with the number of medications consumed. In this population sample, gender did not behave as a predictor variable; however, age was related to severe sarcopaenia. Thus, for each additional year of life, the odds of presenting severe sarcopaenia increased by 10% (OR = 1.11, 95% CI = 1.01–1.22).

## 4. Discussion

The results of the present study show that sarcopaenia (assessed using the EWGSOP algorithm) is present in about a quarter of community-dwelling older patients (sarcopaenia in 20.4% and severe sarcopaenia in 6%). These prevalence data are slightly higher than those reported by similar studies conducted in Spain [27] or elsewhere [28] and are close to the upper limit of the expected range in this health setting. Systematic reviews of studies also carried out on elderly outpatient populations, using the same diagnostic criteria, have reported prevalences ranging from 9.9–40.4% [13], 1–33 % [10], and 10–27% [37]. This considerable heterogeneity between the studies may reflect differences in the diagnostic criteria used, in the cut-off points chosen, and in the characteristics of the target population. In our study, the EWGSOP algorithm was used because it was the working definition most commonly employed when the study began, and thus provided the best comparability with previous work in this area. Moreover, the EWGSOP operational definition offered cut-off points for muscle strength that corresponded to those of the weakness item in the Fried criteria. Very recent studies have shown that the EWGSOP2 diagnostic criteria detect lower prevalences than EWGSOP [38], i.e., the 2010 original version presents greater sensitivity [39].

According to our findings, sarcopaenia is positively associated with age and with the presence of one or more psychopathologies, and inversely correlated with BMI. In our study population, the prevalence of sarcopaenia was higher in women than in men, but a statistically significant association with gender was not confirmed in the multivariate regression model. Although some studies have observed a higher prevalence in the female population [40], most systematic reviews report that more men than women are affected by sarcopaenia [11,14]. There is no clear explanation in this regard, nor has this conclusion been definitively established. It has been suggested that the cut-off value threshold could influence the question [41], or that reduced functional status in men is more closely related to the loss of muscle mass, while in women this decline would be more associated with osteoarthritis, osteoporosis, or depression [42]. We did find a significant relationship with age, such that for each additional year of life, the odds of presenting severe sarcopenia increased by 10%. This is a biologically plausible result that is consistent with previous findings [40,43,44].

Regarding comorbidities, the clinical condition that was associated with all states of sarcopaenia was the diagnosis of psychopathology (mainly anxiety and depression), which doubled the odds of a patient presenting pre-sarcopaenia or sarcopaenia and multiplied them by seven for severe sarcopaenia. Sarcopaenia has most frequently been associated with other chronic conditions such as chronic lung disease, neurological disease, and neoplasia. However, some evidence of a relationship with depression has also been observed, but this association appears to be weaker and is less commonly reported [45,46,47]. An association has also been reported between mental pathology and frailty [48,49]. It has been observed that persons with a psychopathology tend to be less physically active, to have a less active social life, and to consume a less healthy diet, and that any or all of these factors could be related to a loss of strength and muscle mass.

Our findings show that after adjusting for potential confounders, BMI is closely associated with sarcopaenia. Thus, the odds of a patient presenting sarcopaenia and severe sarcopaenia decrease by 27% and 25% for each additional point (kg/m^2^) of BMI, respectively. In consequence, we found the prevalence of sarcopaenia among those with obesity to be significantly lower than among the non-obese population (13.8% vs. 37%, respectively). This inverse relationship between BMI and sarcopaenia is consistent with other studies [43,44]. However, although BMI has been considered an approximate marker of nutritional status, sarcopaenia sometimes coexists with obesity. In our study sample, the prevalence of sarcopaenic obesity was 6.3%, an intermediate figure according to data from a recent systematic review and meta-analysis (2–9%) [37]. It seems that adipose inflammation leads to intra-abdominal fat redistribution and fat infiltration in the muscle. Accordingly, synergy between the loss of muscle mass and this fat infiltration could trigger the pathogenesis of sarcopaenic obesity [50]. In any case, the nutritional status of our community-dwelling older persons was very good (only 1.2% malnutrition), but the presence of obesity was high compared to previous reports. Thus, a study of older adults in 21 European countries reported only 20.9% obesity compared to 45.6% in our sample population [51]. Among other causes, this high prevalence could be due to a certain north-south gradient. According to a national study conducted in Spain, obesity is higher in the south (where Malaga is located) than in the rest of the country [52].

The coexistence of frailty and sarcopaenia was observed in 7.8% of the patients in our study. This rate is lower than that found in another multicentre study conducted in Spain, but the latter focused on hospitalised patients with a higher disease burden, which would explain the discrepancy (18%) [43]. In a recent cohort study of community-dwelling older adults in Australia, more similar to ours, only 2.8% of participants were both frail and sarcopaenic. Among these participants, with either condition, the risk of mortality was over three times higher [53]. The prevalence of frailty among those with sarcopaenia was 40.2%, and that of sarcopaenia among frail patients was 36.6%, results very similar to those found by Reijinierse et al. (42.1% and 36.4%, respectively) who concluded that outpatients with sarcopaenia were more likely to be frail than frail outpatients to be sarcopaenic [54]. Therefore, although sarcopaenia and frailty are related processes and indeed there is some overlap between the criteria that define them, the combined prevalence is low, which reflects the fact that they are different constructs and represent different types of pathophysiology. Sarcopaenia consists of impaired function and muscle mass, while frailty is a broader, multifactorial process that reduces homeostatic reserves. This slight degree of concordance corroborates the conclusions of previous research, in which the two diagnoses did not always coincide according to all definitions applied [54]. In addition, our results show that the intensity of the association between sarcopaenia and frailty was only weak-moderate (Cramer V = 0.45), a low intercorrelation previously reported by the Toledo Study of Healthy Aging (Cramer V = 0.16) [27]. Therefore, it is important to diagnose these conditions separately in order to perform the most appropriate intervention. In accordance with Thompson et al., we believe that individuals identified as frail would benefit from an assessment for sarcopaenia, and vice versa, as a joint assessment is more predictive of mortality than one of either condition alone [53].

It seems well established that progressive resistance training and an adequate protein intake help build muscle mass. In this respect, too, certain dietary interventions (mainly concerning amino acids, vitamin D, antioxidants, and other supplements) are currently being considered [55]. However, although sarcopaenia is currently a topic of great interest, some authors have drawn attention to the possible adverse effects of overdiagnosis and of classifying this phenomenon as a disease. Indeed, it has not been shown that diagnosing sarcopaenia reduces morbidity and mortality, or that the specific treatment for this condition produces better results than the general recommendations of appropriate physical exercise and diet. Moreover, the diagnostic criteria applied tend to be varied and even arbitrary [56]. In view of these considerations, we believe that while sarcopaenia screening studies are positive, encouraging awareness of this condition, revealing its impact and underlining the necessity to adopt an appropriate lifestyle and diet, nevertheless unnecessary labelling should be avoided, and more and better evidence should be obtained about sarcopaenia and its impact on the elderly population.

The study we describe has various strengths. It is based on the analysis of data obtained from a representative sample of healthcare centres and on the global assessment of the participants. The data considered are sufficient in quantity and quality, having been collected directly via personal interviews, anthropometric tests, and medical records. Moreover, in our opinion, the outpatient setting is ideal for assessing conditions such as frailty and sarcopaenia because it is where large numbers of elderly patients are attended and where certain interventions are best performed. Among other strong points of our analysis, the EWGSOP diagnostic criteria were rigorously applied, and the cut-off points used to classify low muscle mass coincide with those of many other studies [40,41,44,57,58]. This parameter was assessed using BIA, as in most studies in the field, due to its accessibility, ease of use, and portability within the health centre. Although DXA is a more precise method, its use in clinical routine is limited by cost considerations and the need for more specialised equipment and personnel. In addition, data suggest there is a good correlation between BIA and DXA [59]. Among the limitations of the study are its cross-sectional design, which means that causal relationships cannot be established, and the fact that it was carried out in a single region and country, which reduces its external validity. However, we believe that the sample considered is representative of a large proportion of community-dwelling older adults, and that the findings found provide a good reflection of circumstances in similar socio-sanitary environments. Another possible limitation of the study is not having considered among the exclusion criteria possible cachexia status, such as cancer and COPD, which are circumstances that can also cause muscle loss.

## 5. Conclusions

According to the EWGSOP criteria, sarcopaenia is a common condition among community-dwelling older persons and may be associated with factors such as age, body mass index, and the presence of one or more psychopathologies. The latter predictive factor may be modifiable or treatable, and thus constitutes a possible area of intervention. Therefore, more attention should be paid to certain signs (or symptoms) to better detect anxiety and depression in the elderly, as these processes tend to be underdiagnosed and appropriate remedial measures would promote healthy aging. Sarcopaenia and frailty are related but separate conditions and require specific approaches.

## Figures and Tables

**Table 1:** Characteristics of the study population (*n* = 333).

Quantitative Variables	Mean	Standard Deviation
Age (years)	72.8	5.1
Lawton (IADL)	6.7	1.7
BMI (kg/m^2^)	30.3	4.9
Number of comorbidities	7.4	3.4
Number of drugs per patient	6.5	4
**Qualitative Variables**	**Subjects (*n*)**	**Percentage (%)**
Gender		
Male	138	41.4
Female	195	58.6
Lawton (IADL)		
0–1	4	1.2
2–3	19	5.7
4–5	44	13.2
6–7	95	28.5
8	171	51.4
SPMSQ (Pfeiffer)		
0–2 errors	305	91.6
3–4 errors	21	6.3
5 errors and over	7	2.1
GDS-15		
0–5	255	76.6
6–9	56	16.8
10 and over	22	6.6
BMI categories		
Underweight	0	0
Normal	49	14.7
Overweight	132	39.6
Obese	152	45.6
Nutritional status		
Normal	318	95.5
Malnutrition risk	11	3.3
Malnourished	4	1.2
Charlson Comorbidity Index		
0–1	219	65.8
2	53	15.9
3 or more	61	18.3
Specific comorbidities		
Bone and joint disorders	256	76.9
Hypertension	227	68.2
Dyslipidaemia	172	51.7
Insomnia	142	42.6
Psychopathology	126	37.8
Diabetes mellitus	89	26.8
Heart disease	81	24.3
Respiratory disease	125	21.5
Osteoporosis	57	17.1
Polymedication	219	65.8
Frailty states		
Robust (non-frail)	72	21.6
Pre-frail	190	57.1
Frail	71	21.3
Fried criterion		
Unintentional weight loss	22	6.6
Exhaustion	68	20.4
Weakness	209	62.8
Slow walking speed	59	17.7
Low physical activity	164	49,2

IADL: Instrumental activities of daily living; BMI: Body mass index (0.0–18.5: underweight; 18.5–24.9: normal; 25.0–29.9: overweight; 30 and over: obese); SPMSQ: Short Portable Mental Status Questionnaire (0–2 errors: normal mental functioning; 3–4 errors: mild cognitive impairment; 5 errors and over: moderate-severe cognitive impairment); GDS-15: Geriatric Depression Scale (0–5: no depression; 6–9: suggestive of depression; 10 and over: almost always depression).

**Table 2:** Sarcopaenia categories and criteria according to EWGSOP (*n* = 333).

	Subjects (*n*)	Percentage (%)
**Sarcopaenia categories**		
No sarcopaenia	192	57.7
Pre-sarcopaenia	53	15.9
Sarcopaenia	68	20.4
Severe sarcopaenia	20	6
**Criteria**		
Low muscle mass	141	42.3
Low muscle strength	209	62.8
Slow walking speed	59	17.7

Categories: No sarcopaenia: 0 criteria present; Pre-sarcopaenia: low muscle mass alone; Sarcopaenia (2 criteria present): low muscle mass + low muscle strength or low performance; Severe sarcopaenia: 3 criteria present.

**Table 3:** Factors related to sarcopaenia. Multinomial logistic regression for pre-sarcopaenia, sarcopaenia, and severe sarcopaenia states (with respect to non-sarcopenic).

Independent Variable	Pre-Sarcopaenia OR (95% CI)	Sarcopaenia OR (95% CI)	Severe Sarcopaenia OR (95% CI)
**Age**	0.94 (0.87–1.02)	0.98 (0.92–1.04)	1.11 (1.01–1.22) *
**Number of comorbidities**	0.79 (0.65–0.97) *	0.92 (0.78–1.08)	1.04 (0.82–1.33)
**Number of medicines**	1.08 (0.95–1.24)	0.98 (0.87–1.10)	1.03 (0.85–1.24)
**BMI**	0.74 (0.67–0.83) ***	0.73 (0.67–0.0.80) ***	0.75 (0.66–0.86) ***
**Gender**			
Male	0.51 (0.22–1.18)	0.64 (0.31–1.31)	0.46 (0.11–1.84)
Female	1	1	1
**Diabetes mellitus**			
Yes	1.12 (0.45–2.76)	0.76 (0.33–1.74)	2.58 (0.79–8.41)
No	1	1	1
**Heart disease**			
Yes	1.04 (0.34–3.09)	1.09 (0.43–2.75)	0.44 (0.89–2.24)
No	1	1	1
**Bone and joint disorder**			
Yes	0.38 (0.16–0.89) *	0.73 (0.32–1.65)	1.59 (0.16–15.09)
No	1	1	1
**Osteoporosis**			
Yes	0.87 (0.31–2.45)	0.28 (0.10–0.79) *	1.10 (0.34–3.58)
No	1	1	1
**Psychopathology**			
Yes	2.56 (1.06– 6.19) *	2.72 (1.30–5.70) **	7.89 (2.25–27.59) ***
No	1	1	1
**Low physical activity**			
Yes	0.31 (0.13–0.72) *	0.95 (0.48–1.85)	1.90 (0.59–6.09)
No	1	1	1

OR: odds ratio; BMI: Body mass index (kg/m^2^). * *p* < 0.05; ** *p* < 0.01; *** *p* < 0.001.

## Data Availability

The data that support the findings of this study are not publicly available due to being used for further investigational objectives and because the data contain information that could compromise the privacy of research participants. However, specific information can be obtained from the corresponding author upon reasonable request (E.B.-R.).
